# Multimodality Imaging in Apical Hypertrophic Cardiomyopathy: Can Echocardiography Learn from Cardiac Magnetic Resonance?

**DOI:** 10.3390/diagnostics15233013

**Published:** 2025-11-26

**Authors:** Francesco Mangini, Massimo Grimaldi, Francesco Spinelli, Santo Dellegrottaglie, Antonio Di Monaco, Simona Quarta, Grazia Casavecchia, Matteo Gravina, Vincenzo Bellomo, Luca Sgarra, Sergio Suma, Gaetano Citarelli, Enrica Filograna, Robert W. W. Biederman, Roberto Calbi

**Affiliations:** 1 Cardiology Department, General Regional Hospital F. Miulli, 70021 Acquaviva delle Fonti, Italy; m.grimaldi@miulli.it (M.G.); a.dimonaco@miulli.it (A.D.M.); v.bellomo@miulli.it (V.B.); l.sgarra@miulli.it (L.S.); 2 Radiology Department, General Regional Hospital F. Miulli, 70021 Acquaviva delle Fonti, Italy; dottorfranco@libero.it (F.S.); r.calbi@miulli.it (R.C.); 3 Cardiology Department, Ospedale Accreditato Villa dei Fiori, 80011 Acerra, Italy; sandel74@hotmail.com; 4 Cardiology Department, Ospedale Policlinico di Bari, Università degli Studi di Bari, 70121 Bari, Italy; quarta_simona@libero.it; 5 Cardiology Department, Ospedali Riuniti Foggia, 71122 Foggia, Italy; graziacasavecchia@libero.it; 6 Radiology Department, Ospedali Riuniti Foggia, 71122 Foggia, Italy; matteo.gravina@unifg.it; 7 Cardiology Department, Azienda Ospedaliero Universitaria, 43126 Parma, Italy; sergiosuma.md@gmail.com; 8 Cardiology Section, Hospital “S. Paolo”, 70123 Bari, Italy; gaetanocitarelli8@gmail.com; 9 Imaging Diagnostics Center, 73048 Nardò, Italy; enricafilograna@gmail.com; 10 Department of Cardiology, Roper/St Francis Hospital, Charleston, SC 29414, USA; robert.biederman1@comcast.net

**Keywords:** cardiomyopathies, apical hypertrophic cardiomyopathy, multimodality imaging, echocardiography, cardiac magnetic resonance imaging

## Abstract

Apical hypertrophic cardiomyopathy is a distinctive and often under-recognized variant of hypertrophic cardiomyopathy, characterized by predominant thickening of the apical segments of the left ventricle. Echocardiography and cardiac magnetic resonance imaging represent the two principal modalities for diagnosis and morphological assessment. While transthoracic echocardiography remains the first-line imaging technique, it may underestimate apical involvement, particularly when image foreshortening or poor endocardial/epicardial delineation occurs. Cardiac magnetic resonance has become the reference standard for defining apical morphology, quantifying hypertrophy, and characterizing myocardial tissue and perfusion. Beyond its diagnostic role, magnetic resonance serves as a research platform for the identification of new apical-centric criteria which, after appropriate validation, may be translated into echocardiographic practice. Echocardiography, however, retains unique strengths through its real-time evaluation of cardiac dynamics, ready-to-use approach to diastolic function assessment, and its ability to identify subtle apical or para-apical obstructive gradients that may raise the initial diagnostic suspicion. This review underscores the complementary roles of the two modalities and the multiple domains in which transthoracic echocardiography can derive substantial methodological and conceptual benefit from cardiac magnetic resonance imaging, both in imaging methodology and in the refinement of diagnostic evaluation.

## 1. Introduction

Hypertrophic cardiomyopathy (HCM) is the most common genetically determined cardiomyopathy, affecting approximately 1/500 to 1/200 of the general population [1]. It is characterized by an increase in the wall thickness of the left ventricle (LV) that cannot be explained by conditions of hemodynamic overload. In most cases, it results from pathogenic mutations in sarcomeric genes. Current diagnostic criteria for hypertrophic cardiomyopathy rely on cardiac imaging–based wall thickness assessment, defining the condition by a maximal left ventricular wall thickness ≥ 15 mm, or ≥13 mm in the context of a positive family history [2]. From a pathogenetic perspective, hypertrophic cardiomyopathy (HCM) encompasses more than the macroscopically defined myocardial hypertrophy, extending to a spectrum of microscopically detectable abnormalities including myocyte disarray, interstitial fibrosis, microvascular dysfunction, and alterations in energy metabolism. These pathological changes collectively contribute to the remodeling of the left ventricle (LV), affecting both its mechanical performance and electrical stability [3]. The concepts outlined above, together with the intricate interplay of genetic, epigenetic, and environmental determinants, collectively shape the profound pathogenic complexity of HCM, a complexity that ultimately manifests as striking clinical heterogeneity, with some individuals remaining asymptomatic for decades, while others develop exertional dyspnea, angina, palpitations, or syncope, and a susceptible minority progressing to sudden cardiac death [4]. Compared to the past, the prognosis of HCM has improved due to more structured clinical pathways, increased awareness of the need for family screening, and, most notably, a more extensive use of multimodality imaging [1,5,6,7]. There are several phenotypic variants of HCM, distinguished by the location and degree of wall thickening. Among these, the apical HCM (ApHCM) is a distinctive subtype, but also the most frequently underdiagnosed. This review critically explores the role of imaging in the diagnosis and management of the apical variant, focusing on the two most commonly used methods in clinical practice and recommended for this pathology: transthoracic echocardiography (TTE) and cardiac magnetic resonance imaging (CMRi). Additionally, it illustrates the different possible domains in which TTE can derive substantial methodological and conceptual benefit from CMRi, both in terms of imaging methodology and in the refinement of diagnostic evaluation.

## 2. Overview of ApHCM

Within the phenotypic spectrum, ApHCM represents a variant first described in the late 1970s by Yamaguchi [8], now explicitly recognized in a recent EACVI Clinical Consensus as a distinct morphological subtype [9]. Typical and classically described features include the presence of deep negative T waves on the electrocardiogram (ECG) [10], apical wall thickening with obliteration during systole, and in more advanced forms, the development of an apical aneurysm [11,12]. Epidemiology varies according to ethnicity and imaging methodology: in Asian cohorts, the proportion of ApHCM among all HCM can reach 25% [6,13], whereas in Europe and North America, it is more often between 1 and 10% [6,14,15]. For a long time, ApHCM was considered a relatively “benign” variant [14]. However, recent evidence over the past decade has updated this perception: atrial dilation, myocardial fibrosis, apical aneurysm, and mid-ventricular systolic obstruction are elements that influence risk and explain less reassuring clinical trajectories in specific subgroups [12,16,17], with an overall mortality rate reported between 0.5% and 3.5% [6]. Guo et al. recommend distinguishing the overall apical phenotype from its risk subgroups, providing a less stereotypical and more clinically relevant picture; indeed, in a study on a Chinese cohort of nearly 500 patients, including over 100 with ApHCM, it has been shown that, compared to non-apical forms, ApHCM patients are generally older at diagnosis, show higher ejection fraction (EF), and a lower long-term risk of events; within the apical phenotype, age and EF remain independent predictors of outcomes [6]. In diagnostic workup, TTE is the first-line method, while advanced imaging involves CMRi, which remains the gold standard for morphological study and tissue characterization, with complementary roles for CT and nuclear imaging [18,19,20,21]. In septal phenotypes, the presence of a dynamic gradient in left ventricular outflow tract (LVOT) has a well-established prognostic significance and guides treatment options such as myectomy or septal alcohol ablation in cases refractory to medication [22]. In ApHCM, the dynamic of obstruction is generally not localized to the LVOT but occurs in the mid-cavity and apex, where mid-ventricular systolic obstruction compartmentalizes the ventricle into proximally and distally separated hemodynamic regions, increasing apical pressure and predisposing to aneurysm formation [16]. The relationship between mid-ventricular systolic obstruction and aneurysm has been frequently documented in dedicated case series [12]. From an electrocardiographic, morphological, and tissue characterization perspective, the apical variant exhibits typical features such as deep, sometimes giant negative T waves [10], the LV cavity can assume the characteristic “sword-shaped” profile during systole, and fibrous substitution with a typical pattern often more intense at the apex, along with left atrial remodeling [2,23,24]. In normal hearts, basal segments are thicker than apical ones; therefore, reaching the conventional 15 mm cutoff requires a greater degree of thickening in the apical segments than in the basal regions. Consequently, universally applying a 15 mm threshold regardless of the segment considered may not be appropriate. Indeed, there is still no consensus on the definition of ApHCM, and some true cases of apical involvement may go unrecognized. In clinical practice, ECG alterations and underdiagnosis via TTE often lead clinicians to suspect ischemic etiology, prompting further investigation with CMRi after ruling out coronary artery disease. Beyond the mere apical wall thickening, novel criteria have been proposed, mostly based on CMRi studies, which may increase the likelihood of identifying this pathology through imaging [25]. The main epidemiological, electrocardiographic, and imaging features of classic versus apical HCM are summarized in Table 1. Recent evidence highlights the importance of distinguishing between pure-apical and distal-dominant forms of ApHCM, as they likely represent distinct clinical and genetic entities with different morphological evolution and prognostic implications. In classic ApHCM, hypertrophy is typically confined to the apex below the level of the papillary muscles; however, particularly in Western populations, a distal-dominant variant has been described, characterized by wall thickening that extends to the midventricular segments, most frequently the midventricular septum, thereby blurring the anatomical boundary between apical and midventricular phenotypes. Sugiura et al. [26] recently demonstrated that patients with distal-dominant ApHCM show a higher prevalence of sarcomeric mutations, greater apical wall stress, and a more adverse arrhythmic substrate compared with pure-apical forms, highlighting the clinical relevance of refined imaging-based phenotyping.

## 3. Multimodal Imaging: TTE and CMRi as Main Actors

The recent European and American guidelines explicitly recommend a multimodal approach for the assessment of HCM [2,22]. A recent review describes the role of different methodologies, including computed tomography (CT) and nuclear medicine [18]. Currently, in clinical practice, the two most used examinations are TTE and CMRi. TTE is the initial examination due to its wide availability, relatively short duration, and repeatability. CMRi represents the standard for morphological and kinetic studies of the walls, for accurate quantification of volumes and ventricular mass [18,19,27,28]; additionally, CMRi is today the gold standard for tissue characterization (LGE for fibrotic replacement, T1/T2 mapping and ECV for diffuse fibrosis/edema). CT and nuclear imaging can also play a role. Cardiac CT, especially in dual-energy or spectral iterations, allows high-quality cine-morphology and, in selected cases, tissue characterization via “late iodine enhancement” sequences as a surrogate for scar tissue when CMRi is contraindicated. Single photon emission computed tomography (SPECT) and positron emission tomography (PET) perfusion imaging add a pathophysiological dimension: ApHCM can display a characteristic apical perfusion pattern (the so-called “solar polar” on SPECT), while stress PET quantifies a selective reduction in apical flow consistent with the “almost universal apical ischemia” observed in CMRi studies [18,19]. However, because patients are not typically referred for second-level imaging unless suspicion is first raised on TTE, it is essential, indeed, the central purpose of this review, that echocardiography be able at least to identify those cases in which ApHCM should be suspected.

## 4. Echocardiography

**Figure 1:**
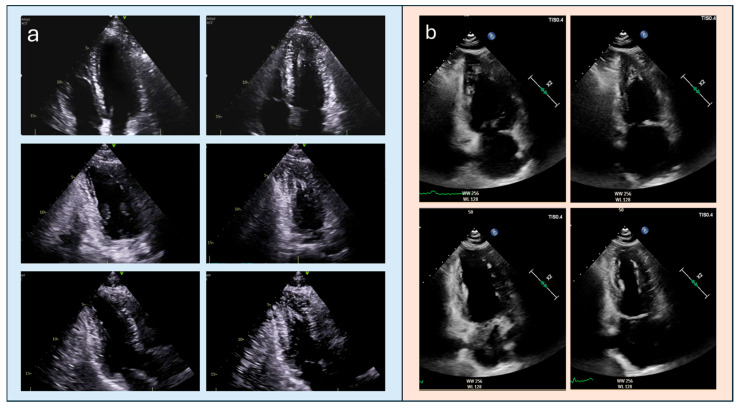
TTE assessment of long-axis views. (**a**): TTE long-axis views in a patient with ApHCM: correctly acquired apical four-, two-, and three-chamber views at end-diastole (right) and end-systole (left), demonstrating appropriate visualization of the true apex. (**b**) TTE long-axis views in a normal patient: example of apical foreshortening in the apical two-chamber view (upper row), which may falsely suggest apical hypertrophy; the correctly obtained view (lower row) restores the true apical geometry and demonstrates normal apical morphology. TTE, transthoracic echocardiography; ApHCM, apical hypertrophic cardiomyopathy.

**Figure 2:**
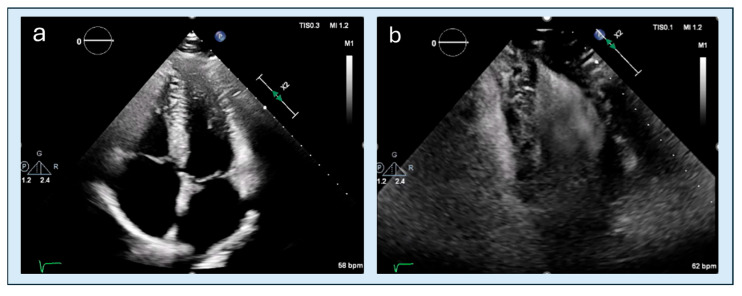
(**a**) Apical four-chamber echocardiographic view without the use of an intracavitary contrast agent. (**b**) Apical four-chamber echocardiographic view with the use of an intracavitary contrast agent. Contrast enhancement markedly improves endocardial border delineation and reveals the characteristic “spade-like” apical configuration, which is poorly defined on unenhanced imaging.

TTE is the first line imaging examination in most settings [1,6]. Its strength lies in availability and in dynamic assessment, i.e., the ability to measure gradients and capture physiological and hemodynamic phenomena in real-time; its limitations include intra/inter-operator variability, a tendency to underestimate volumes and overestimate EF compared to CMRi and other second-level methods, and a lower signal-to-noise ratio, which limits morphological evaluation in some contexts [24]. A general limitation of TTE in wall thickness assessment is the reduced signal-to-noise ratio at the blood–endocardium interface, compared to CMRi [7], which becomes particularly relevant at the apex; at this level, there is typically a greater trabecular expression and a relatively reduced cavity; these elements cause endocardial delineation to be frequently suboptimal, and wall thickness is systematically underestimated unless contrast is employed. Another limitation lies in the execution of the exam, where the most insidious error is the foreshortening of apical views: a window that is too high artificially shortens the ventricle, reduces volumes, alters the EF estimate, and, most importantly, can make the true apex ‘disappear,’ replacing it with a fictitious apex. The two possible consequences are either an overestimation of the wall thickness at the apex due to signal overlap or an underestimation of apical hypertrophy with distortion of strain measurements [29]. Hence, it is important to maximize the longitudinal extension of the LV in views 2, 3, and 4 chambers and to carefully check the probe orientation (Figure 1). The parasternal long axis remains the starting point for measuring septal and posterior wall thicknesses in diastole, with the beam perpendicular to the septum to avoid systematic errors [30]. Optimizing parameters is crucial: excessively high gain can generate artifacts in the cavity and simulate an apical obliteration that does not exist; too low gain can erase the endocardial profile precisely where it needs to be delineated better, namely at the apex margins. A balanced setting allows distinguishing a hypokinetic apex with residual cavity from a truly hypertrophic and obliterated apex in systole [31]. It is advisable to consider long-axis views, specifically the apical four-, two-, and three-chamber views as the primary reference for measuring left ventricular wall thickness, as they provide optimal alignment with the ventricular axis and a more reliable depiction of apical segment morphology. Whenever feasible, these measurements should be corroborated by short-axis views, which often allow clearer visualization of trabeculations and help distinguish true hypertrophy from trabecular interference. This combined approach ensures a more accurate and anatomically robust assessment of all left ventricular segments. When endocardial delineation is imperfect in two or more segments, the EACVI/ASE recommendations suggest using contrast media [32]. In ApHCM, contrast usually highlights the ‘spade’ profile clearly (Figure 2) and more generally allows detection of aneurysms and thrombi that unenhanced TTE might miss; a clinical-imaging review from 2025 reaffirmed that TTE, if not rigorously performed or without contrast, may fail to detect apical aneurysm in up to 43% of patients [20]. Transesophageal echocardiography, although useful in septal forms for studying the left ventricular outflow tract (LVOT) and the mechanism and grading of mitral regurgitation, generally has limited utility in the apical variant: the difficulty in visualizing the apex with TEE and the low frequency of mitral anomalies in pure forms reduce the clinical impact of this technique in ApHCM.

### 4.1. Obstruction

**Figure 3:**
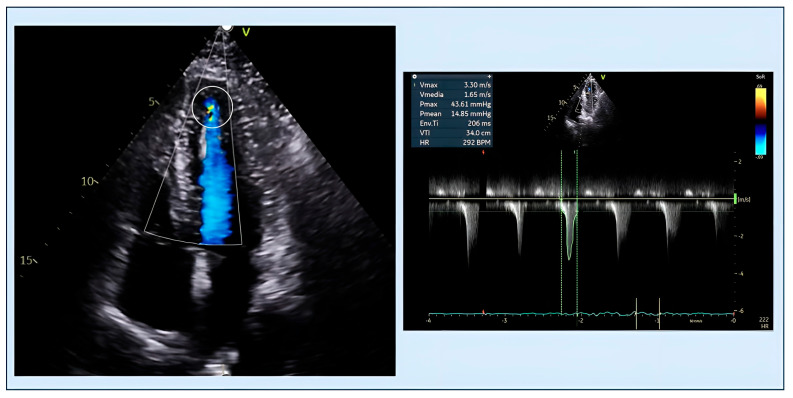
A 38-year-old male patient with ApHCM. Color Doppler imaging shows apical aliasing artifact (circled), suggesting localized obstruction. Continuous-wave Doppler aligned with the same jet confirms an apical systolic gradient, consistent with obstruction in ApHCM. ApHCM, Apical hypertrophic cardiomyopathy.

One of the strengths of echocardiography is real-time dynamic assessment. Provocative maneuvers such as the Valsalva maneuver, squat-to-stand, and even the simple post-prandial state can reveal latent dynamic gradients at the LVOT and, in ApHCM, especially in the mid-cavity region [33]. When obstruction is marked, continuous Doppler reveals a ‘mid-systolic signal-void,’ a black zone in the CW trace indicating transient cessation of flow during mid-systole, effectively indicating compartmentalization of the LV [34]. In rarer cases, obstruction may localize at the level of the purely apical cavity (Figure 3); since normally no gradients are expected at the apex, this finding should raise suspicion of ApHCM, regardless of the absolute wall thickness. Because in ApHCM the relevant hemodynamics occur away from the outflow, systematic investigation for mid-ventricular obstruction is crucial: aligning the beam along the cavity axis, ‘catching’ the apical jet, and quantifying the gradient are essential steps in a laboratory truly interested in this phenotype. A practical echocardiographic issue is distinguishing truly mid-ventricular obstruction (MVO) from mere changes in the CW Doppler configuration recorded at a similar mid-ventricular level in the presence of left ventricular outflow tract obstruction (LVOTO). In true mid-ventricular obstruction (this concept also applies to apical obstruction), two-dimensional imaging typically shows systolic “hour-glass” cavity narrowing at the mid-ventricular level, with color aliasing originating at this site and CW Doppler demonstrating a high-velocity jet (usually ≈2.5–4.0 m/s) and late-peaking or even double-peaked envelopes that may extend into isovolumic relaxation or early diastole, in keeping with a persistent apex-to-base intraventricular gradient [35]. By contrast, in LVOTO with only mid-ventricular flow configuration changes (for example due to trabeculation, apical remodeling, or cavity tapering), the principal obstruction and highest velocities are confined to the subaortic outflow tract, yielding the classic dagger-shaped late-systolic CW profile at the LVOT, whereas any mid-ventricular signal is of lower velocity, strictly systolic, and not associated with clear mid-cavity obliteration or diastolic apex-to-base flow reversal [36].

### 4.2. Apical Obliteration

**Figure 4:**
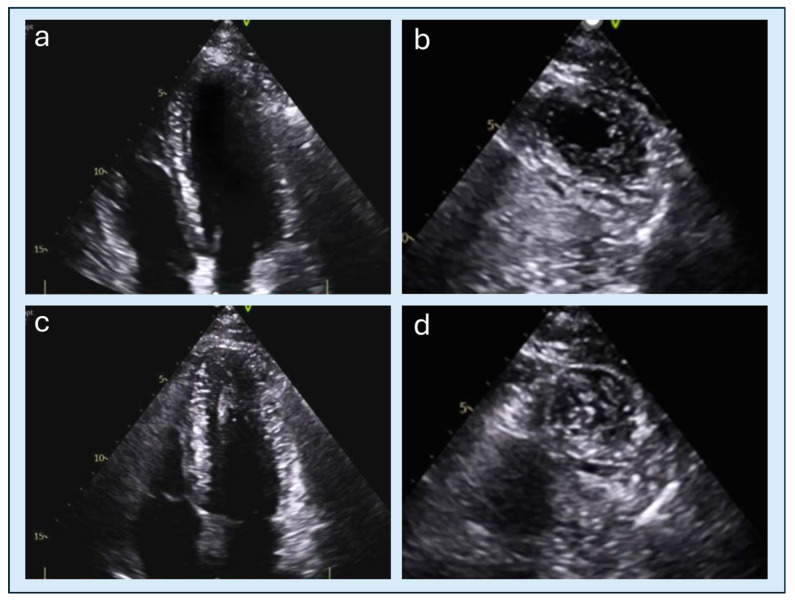
TTE assessment of apical obliteration. (**a**) apical four-chamber long-axis view in diastole, showing a preserved apical cavity. (**b**) focused short-axis view in diastole, confirming a residual apical lumen. (**c**) apical four-chamber long-axis view in systole, demonstrating near-complete obliteration of the apical cavity. (**d**) focused short-axis section in systole, showing circular endocardial apposition consistent with apical obliteration.

Apical obliteration represents one of the characteristic morphologic features of ApHCM and reflects the extreme systolic apposition of the apical walls, often accompanied by mid-ventricular cavity compartmentalization. The reference views for identifying apical obliteration are the apical long-axis views (Figure 1); however, performing a focused short-axis section on the apex can also be helpful in showing apical obliteration, especially when apical sections are insufficient (Figure 4). For many years, its evaluation remained purely qualitative, based on visual identification of complete systolic closure of the apical cavity. More recently, however, attempts have been made to quantify the extent of apical obliteration, introducing geometric ratios and standardized measurements (see the criterion described in Section 9). This quantitative approach allows a more reproducible assessment and may help identify patients at greater risk of adverse events associated with significant apical compartmentalization.

### 4.3. Speckle Tracking Imaging

**Figure 5:**
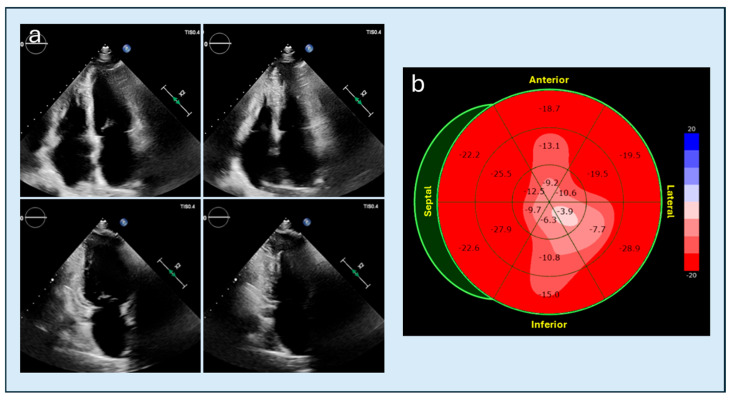
Speckle-tracking echocardiography in a patient with ApHCM. (**a**) apical four- and two-chamber views showing systolic apposition of the apical walls with marked reduction in the apical cavity. (**b**) bull’s-eye longitudinal strain map demonstrating reduced apical strain values compared with mid- and basal segments, simulating reduced contractility and reflecting the mechanical constraint caused by apical segments contracting against each other during systole. ApHCM, apical hypertrophic cardiomyopathy.

Global longitudinal strain (GLS) and segmental strain allow the detection of regional dysfunction even when the EF is normal. In ApHCM, the contraction of apical segments is frequently mechanically constrained by systolic apposition of the apical walls. This phenomenon results in a characteristic pattern of apical “shrinkage” on longitudinal strain analysis, with the bull’s-eye map demonstrating reduced apical strain values relative to the mid- and basal-ventricular segments [37] (Figure 5). Associated with this are alterations in apical rotation and global twist: the apex loses its traditional rotational dominance, and the baso-apical gradient flattens [18]. Myocardial work, integrating strain and blood pressure, promises to offer additional prognostic signals in HCM [38]. Finally, 3D echocardiography helps reduce inter-operator variability and provides more reliable volumetry, especially in apical regions, with automated models that quickly return measurements of mass, shape, volumes, and EF [39]. In this context, a 3D ‘mass dispersion index’ has been proposed as an indicator of hypertrophic asymmetry; it is an intriguing concept validated for HCM but still requiring specific validation for ApHCM [18].

### 4.4. Perfusion

Alongside hemodynamics, a ‘microcirculation’ perspective is emerging with contrast-enhanced perfusion imaging. Low-mechanical-index contrastography, at rest and under stress, can reveal segmental perfusion differences that often favor the apex in ApHCM [40]. This is an evolving field, but convergence with SPECT/PET and CMRi perfusion, which consistently indicate almost universal apical ischemia in the ApHCM phenotype, makes this line of investigation particularly promising [17,19].

## 5. Differential Diagnosis and Interpretative Pitfalls

**Figure 6:**
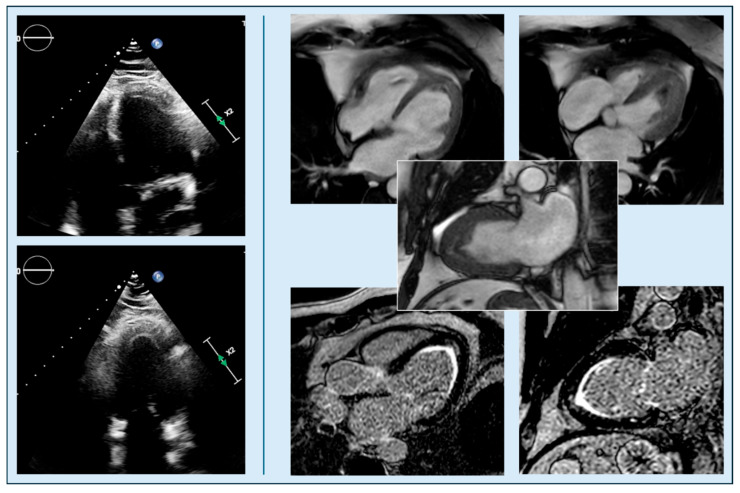
A 41-year-old male patient showing non-specific ECG abnormalities; case initially interpreted as ApHCM on TTE (**left**: apical four-chamber view, top; two-chamber view, bottom), but subsequently identified as eosinophilic myocarditis on CMRi (**right**). In fact, cine CMRi images (upper panels) reveal that the apparent apical thickening is due to non-myocardial material (fibrosis/thrombus), with the characteristic “flying V” sign (central inset). Late gadolinium enhancement sequences (lower panels) confirm the diagnosis, showing diffuse subendocardial enhancement of the mid-to-apical segments, typical of eosinophilic. CMRi, cardiac magnetic resonance imaging; TTE, transthoracic echocardiography, ApHCM, Apical hypertrophic cardiomyopathy.

The first pitfall lies in the failure to recognize the disease itself, leading to underdiagnosis. When wall thickening is detected, a further challenge is represented by the potential difficulty in accurately determining the precise level from which hypertrophy originates. This limitation may hinder a reliable differential diagnosis between the pure-apical and distal-dominant forms of ApHCM. Furthermore, ApHCM shares numerous morphological and functional characteristics with other cardiac conditions that can make differential diagnosis complex, especially when hypertrophy is mild or confined to the apex. Major alternative diagnoses include physiological hypertrophy in athletes, secondary hypertrophy due to pressure or volume overload, non-sarcomeric forms, such as cardiac amyloidosis or Fabry disease. Another condition that can mimic ApHCM is eosinophilic myocarditis, which shares apical involvement with it. In the subacute phase, fibrosis and endocardial thickening produce a pseudo-hypertrophic echocardiographic appearance; CMRi allows differentiation between the two entities thanks to the presence of diffuse subendocardial Late Gadolinium Enhancement (LGE) and segmental edema, findings not typical of ApHCM (Figure 6). An additional diagnostic pitfall is the potential misdiagnosis of apical hypo-akinesia in patients with apical myocardial hypertrophy. The presence of hypertrophic segments contracting against themselves at the apex can cause mechanical limitation of cardiac movement, mimicking hypokinesia (Figure 5). The distinction between these two conditions is crucial: in ApHCM, the reduction in contractility is caused by mechanical limitation and is persistent, often associated with cavity obliteration while hypo-akinesia is usually associated with an increase in the volume of the apical cavity. Furthermore, conditions of absolute or relative hypovolemia and hyperdynamic states can simulate ApHCM due to the tendency for systolic obliteration, which is generally more evident at the apex in these conditions. To provide guidance in clinical scenarios where transthoracic echocardiographic findings prove insufficiently definitive, a modality-specific and progressive interpretative strategy can contribute to a more accurate diagnostic process. When image quality is limited by inadequate acoustic windows or by apical foreshortening, the first step is to repeat the acquisition with meticulous optimization of apical views. If uncertainty persists, contrast-enhanced TTE is particularly helpful because it improves endocardial border delineation and allows a more reliable reassessment of apical wall thickness, tapering and systolic obliteration. If contrast-enhanced TTE still does not clarify whether true apical hypertrophy is present, CMRi represents the subsequent investigation. CMRi is also valuable when apparent apical hypokinesia on TTE may instead reflect mechanical restriction from hypertrophic segments. Cine imaging and strain analysis can help distinguish constrained motion from true systolic impairment. When an infiltrative or inflammatory condition such as amyloidosis, Fabry disease or eosinophilic myocarditis is suspected, CMRi provides essential diagnostic information. In patients presenting with hypovolemia or hyperdynamic states, repeating TTE after hemodynamic stabilization is recommended, because physiological cavity reduction may accentuate apical obliteration and mimic pathological findings. In summary, a rigorous echocardiographic evaluation combined with the selective use of CMRi when uncertainty persists allows clinicians to navigate atypical or complex presentations more confidently and reduces the risk of interpretative pitfalls in the assessment of apical pathology. The subsequent section will address in detail the role and potential of CMRi in this context.

## 6. Cardiac Magnetic Resonance Imaging

In HCM, current ESC and AHA/ACC guidelines recommend CMRi at the initial stage of the diagnostic workup, when tissue characterization is required, particularly in the suspicion of non-sarcomeric forms, when transthoracic echocardiography is inconclusive, or when additional information on fibrosis, aneurysms, thrombi, apical morphology, or perfusion is needed [2,22].

### 6.1. Cine-MR and Morphology

**Figure 7:**
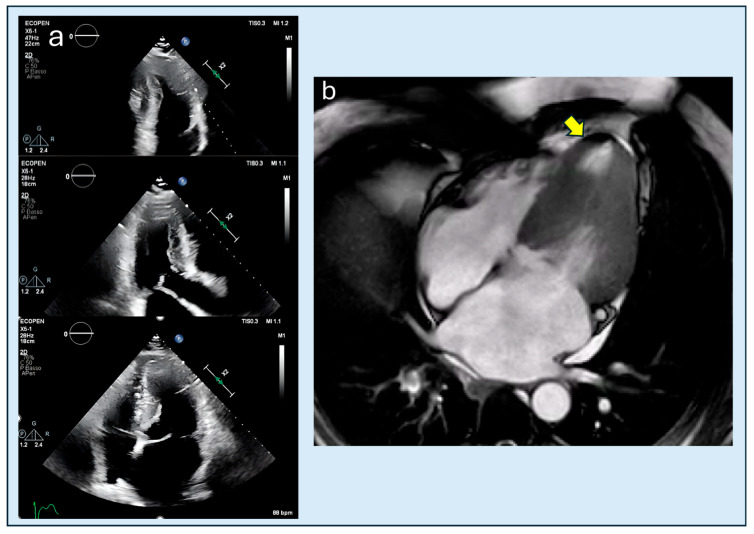
54-year-old male patient with HCM. (**a**) TTE failed to detect the apical aneurysm, likely due to limitations related to suboptimal acoustic windows and reverberation artifact. (**b**) CMRi clearly demonstrates the early aneurysmal remodeling of the apical septum (yellow arrow). HCM, hypertrophic cardiomyopathy; TTE, transthoracic echocardiography; CMRi, cardiac magnetic resonance imaging.

Sequences such as SSFP cine form the basis of morphological and functional assessment: the high signal-to-noise ratio and optimal blood-myocardium contrast allow accurate measurement of volumes, mass, and ventricular function, especially in difficult regions like the apex and lateral wall [41,42]. Compared to TTE, CMRi can uncover “occult” or limited hypertrophies, reliably recognize apical aneurysms (Figure 7), and describe atypical papillary-trabecular geometries (including crypts), all elements supporting diagnosis even when wall thickness does not reach traditional thresholds [9,10,42]. Furthermore, CMRi allows for accurate identification of the level at which apical thickening begins and extends distally and proximally consequently permitting a clearer distinction between pure-apical and distal-dominant variants of ApHCM [26].

In CMRi, obstruction can be identified: areas of high-velocity turbulence generate typical “dephasing” artifacts (signal void) on gradient-echo sequences, serving as visual indicators of obstruction, most often, but not exclusively, at the LVOT [43,44]. Quantitative analysis of the gradient remains complex due to the dynamic geometry of outflow and the limitations of provoking maneuvers within the scanner, which is why stress-dependent hemodynamic quantification remains the domain of TTE. Conversely, CMRi provides a robust assessment of mitral apparatus behavior, including SAM presence and quantification of mitral regurgitation through combined volumetric cine and phase-contrast flow mapping [45].

### 6.2. Feature Tracking and Strain

Myocardial tissue tracking on cine images adds important functional and prognostic information: early reductions in left ventricular and left atrial strain are detectable even when the ejection fraction is still preserved and have been shown to correlate with circulating markers of myocardial injury and wall stress, specifically high-sensitivity cardiac troponins and N-terminal pro–B-type natriuretic peptide. Moreover, alterations in diastolic strain rates have been associated with adverse clinical outcomes, including death from any cause, death related to heart failure, death related to stroke, and documented episodes of exertional or rest-related decompensation. Impaired circumferential strain has also been linked to an increased burden of ventricular arrhythmias, such as non-sustained and sustained ventricular tachycardia, as well as resuscitated cardiac arrest, while non-contrast tissue tracking parameters have emerged as potential indicators of underlying myocardial fibrosis. Advanced techniques, including myocardial tagging and three-dimensional approaches, further improve sensitivity for detecting subtle regional dysfunction and for characterizing remodeling after therapeutic interventions [46,47,48,49]. Advanced techniques like tagging and 3D approaches further extend sensitivity for regional function and post-treatment remodeling.

### 6.3. Tissue Characterization

#### 6.3.1. T1 Mapping and ECV

T1 mapping and extracellular volume (ECV) quantify diffuse interstitial fibrosis and complement LGE, which identifies replacement fibrosis. Inversion recovery-based techniques (MOLLI, ShMOLLI) show high reproducibility and diagnostic sensitivity [50,51,52]. In HCM, native T1 and ECV increase with phenotypic severity, correlate with LV mass and wall thickness, and help differentiate sarcomeric HCM from hypertensive hypertrophy [49,50,51]. Their elevation reflects extracellular expansion from interstitial fibrosis and myocyte disarray. In ApHCM, evidence is more limited, though recent work confirms the diagnostic and prognostic value of parametric mapping without variant-specific cut-offs [53,54]. In mixed HCM cohorts, apical segments show higher native T1 and ECV than basal ones, consistent with greater microvascular vulnerability. T1 mapping also aids differential diagnosis from apical mimickers (e.g., Takotsubo syndrome, eosinophilic myocarditis), where normalized T1/ECV at follow-up indicates transient edema rather than fibrosis [55]. Reporting should include protocol, field strength, vendor, and local reference ranges following EACVI-SCMR recommendations [27,28].

#### 6.3.2. T2 Mapping

T2 mapping quantifies myocardial edema and sensitively detects inflammatory or ischemic activity. In HCM, increased T2 occurs even in non-hypertrophied segments and is associated with reduced strain and systolic thickening [54]. In ApHCM, the pattern is typically apice-centric, reflecting chronic wall stress and distal microvascular dysfunction. Combined elevation of T2 and ECV identifies a transitional phase from reversible edema to emerging fibrosis, offering value for disease monitoring [19,56].

Across all mapping techniques, the apical phenotype presents inherent technical limitations related to increased susceptibility to motion artifacts, partial-volume contamination from trabeculations, and field inhomogeneities, and current evidence remains limited because relatively few mapping data have been specifically validated in apical forms.

#### 6.3.3. Late Gadolinium Enhancement

**Figure 8:**
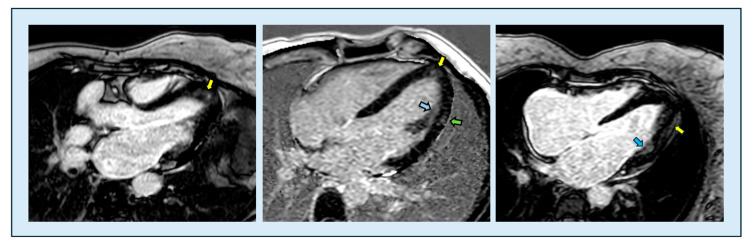
Late gadolinium enhancement sequences in ApHCM. Patchy mid-wall fibrosis is visible within the most hypertrophic apical segments (**left** and **middle** images, yellow arrows), but it may also involve non-hypertrophied regions (**middle** image, green arrow) and papillary muscles (**middle** image, blue arrow). In addition, a subepicardial enhancement pattern (**right** image, yellow arrow) and an ischemic subendocardial pattern (**right** image, blue arrow) have also been described in ApHCM. LGE, late gadolinium enhancement; ApHCM, apical hypertrophic cardiomyopathy.

LGE is the gold standard for assessing replacement fibrosis. After gadolinium administration, inversion-recovery sequences, preferably in PSIR mode (Phase-Sensitive Inversion Recovery), allow nulling of normal myocardium signal and visualization of fibrotic areas [57,58]. Optimal acquisitions involve complete short-axis coverage with voxel sizes around 1.5 mm and a post-contrast delay of 10–15 min. Dark-blood sequences improve endocardial definition and thrombus detection, while wide-band variants reduce artifacts in device carriers [41]. In HCM, replacement fibrosis is detected in more than half of patients, typically exhibiting a patchy mid-wall distribution within hypertrophied myocardial segments; however, other patterns have also been described such as subepicardial or diffuse mid-myocardial fibrosis, whereas a subendocardial pattern has been reported in association with ischemic imbalance due to microvascular dysfunction [18,57,59,60,61]; an extension ≥15% of LV mass strongly predicts ventricular arrhythmias and sudden death [59,60,61,62]. In ApHCM, LGE prevalence ranges from 60% to 80% [11,63]. The pattern is mainly apical or peri-apical, with possible extension to mid-distal septal or inferior wall segments (Figure 8). Yang et al. (2020) reported LGE in 87% of patients with ApHCM and apical aneurysm, compared to 52% without aneurysm; its extent correlated with ventricular arrhythmias and major cardiovascular events [11]. Li et al. (2021) [64] documented a 67% prevalence of LGE in a cohort of ApHCM, with an average extent of 4.9% of LV mass and an independent prognostic value for cardiovascular events when ≥5%. Studies also showed progressive increase in apical fibrotic burden during follow-up, associated with aneurysm development and increased arrhythmic risk [65]. Co-localization of LGE and perfusion defects on CMRI supports the pathophysiological model of microvascular ischemia leading to fibrosis leading to apical aneurysm [19].

### 6.4. Perfusion and Microvascularity

CMRi perfusion is the most sensitive method for detecting microvascular dysfunction, a key aspect of HCM. Rest perfusion defects correlate with mass and wall thickness, while adenosine stress reveals abnormalities in 40–50% of patients, often associated with extensive LGE and non-sustained ventricular tachycardia [66,67]. Myocardial Blood Flow (MBF) and Myocardial Perfusion Reserve (MPR) quantification shows reductions even in non-hypertrophied segments, suggesting microvascular disease may precede overt hypertrophy [68,69]. In ApHCM, microvascular dysfunction is nearly universal, with selective apical flow reduction and heterogeneous perfusion reflecting apico-basal pressure gradients and high apical fibrosis [19,68]. Combined analysis of perfusion, mapping, and LGE helps distinguish reversible ischemia from fibrosis, supporting prognosis and therapy.

#### Intracavitary Dynamics and 4D-Flow

4D-FLOW CMRi enables three-dimensional and temporal representation of intracavitary flow, quantifying vortices, pressure gradients, kinetic energy, and energy losses. In HCM, this technique has demonstrated flow inefficiencies even in the absence of outflow gradients, which correlate with wall deformation and fibrosis [70,71]. In ApHCM, the middle-apical compartmentalization and “funnel-shaped” geometry cause persistent turbulence and vortices, increasing wall stress and energy loss. These phenomena, also detectable with echocardiographic Vector Flow Mapping (VFM), contribute to understanding the relationship between morphology and hydrodynamic inefficiency [72].

The integration of 4D-FLOW, Feature Tracking, and tissue mapping represents the most promising approach for quantitative hemodynamic characterization of ApHCM and for identifying new risk biomarkers.

### 6.5. Practical Limitations and Technological Developments

CMRi is limited by acquisition times, availability, and costs while technical issues include claustrophobia and presence of non-conditional devices. New sequences such as PSIR, dark-blood, wide-band, and accelerated acquisitions with Compressed Sensing have improved sensitivity, resolution, and feasibility of LGE imaging [58]. The integration of Artificial Intelligence and Machine Learning in automatic segmentation, LGE quantification, and 4D-FLOW processing will potentially enable the development of phenotype-specific risk scores based on the integration of morphology, tissue, and function [72,73].

## 7. Diagnosis and Novel Diagnostic Criteria

**Figure 9:**
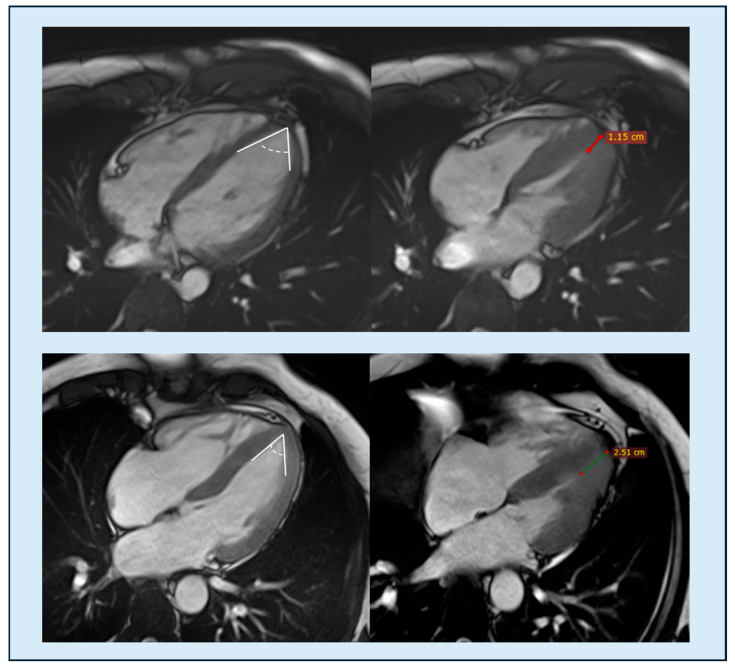
Comparison of two patients with a base-to-apex wall thickness ratio of 1. Top row: patient with hypertensive heart disease showing a normal apical angle (**right**) and limited apical obliteration (**left**) of 11 mm (<20 mm); base-to-apex wall thickness tapering appears preserved. Bottom row: patient with confirmed apical hypertrophic cardiomyopathy (ApHCM), characterized by a reduced apical angle (**left**) and marked apical obliteration (**right**) of 25 mm (>20 mm); base-to-apex wall thickness ratio is approximately 1. ApHCM, apical hypertrophic cardiomyopathy.

**Figure 10:**
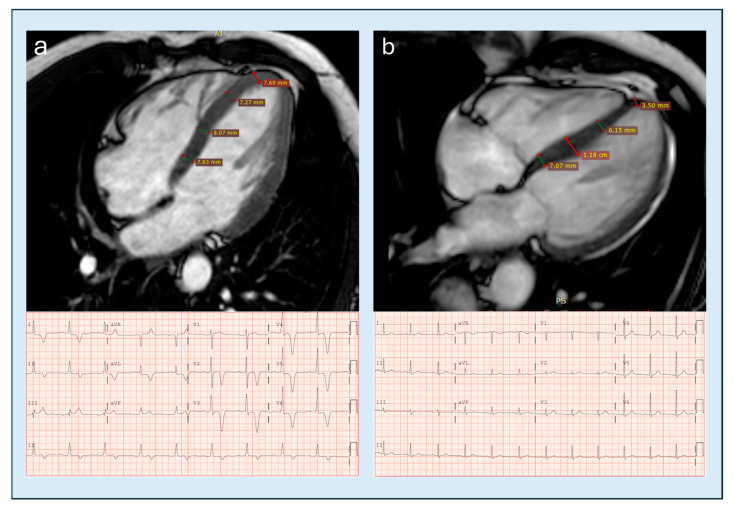
Base-to-apex wall thickness tapering by CMRi and corresponding 12-lead ECG in two different patients. (**a**) a patient with apical hypertrophic cardiomyopathy (ApHCM), showing a maximal apical wall thickness of 8 mm without Base-to-apex tapering, associated with striking electrocardiographic abnormalities, namely deep negative T waves. (**b**) a patient with hypertensive heart disease, with a greater absolute wall thickness (12 mm), preserved basal–apical tapering and a normal ECG finding. CMRi, cardiac magnetic resonance imaging, ApHCM, apical hypertrophic cardiomyopathy; ECG, electrocardiogram.

**Figure 11:**
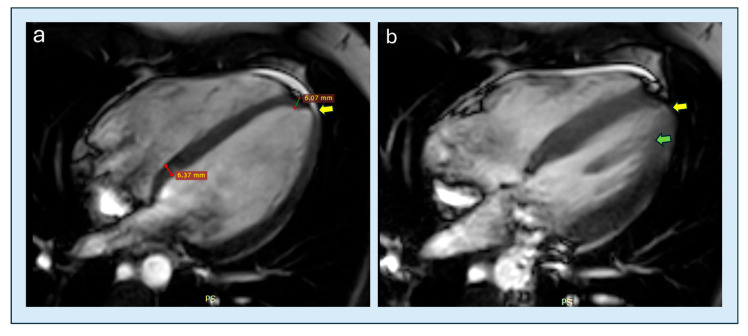
A 49-year-old female patient with documented ApHCM (**a**) diastole: the patient exhibits base-to-apex tapering ratio of approximately 1 and diastolic thinning of the apical cap (yellow arrow). (**b**) systole: the same patient exhibits and an initial, focal tendency toward apical aneurysmal evolution (yellow arrow); in addition, there is apical displacement of the posterolateral papillary muscle (green arrow, right). ApHCM, apical hypertrophic cardiomyopathy.

As also emphasized in the 2025 EACVI Clinical Consensus [9], classical diagnostic thresholds based solely on absolute apical wall thickness (≥15 mm) have limited sensitivity, particularly in early or “relative” ApHCM, where hypertrophy may be subtle or confined to small segments. Over the past decade, several apical-centric diagnostic criteria derived primarily from CMR have improved phenotype recognition and may provide conceptual models to refine echocardiographic assessment. Because these criteria originate from CMR cohorts, understanding their methodological background is essential to evaluate their potential transferability to TTE, which remains the first-line imaging modality. One of the most relevant developments is the body-surface-area–indexed apical thickness proposed by Hughes et al. [24], who demonstrated that an indexed threshold of 5.2–5.6 mm/m^2^ (≈11 mm unindexed) identifies almost all overt and most relative ApHCM cases while maintaining high specificity. Although this index has not yet been validated for echocardiography, it highlights how geometric normalization, widely used in CMR, may guide the future development of standardized TTE cut-offs. Similarly, apical angle measurement, introduced by Li et al. [64], has shown discriminatory value in distinguishing apical from non-apical forms (76° vs. 89°, *p* = 0.016) (Figure 9). Its use in echocardiography is conceptually feasible but limited by the risk of foreshortening, and dedicated acquisition protocols and validation studies are needed before routine application. Other CMR-derived geometric markers exhibit stronger alignment with echocardiographic assessment. The apical-to-basal wall thickness ratio, for example, is traditionally set at >1.3 and more recently considered diagnostic even when >1 [74], can often be evaluated on optimized apical TTE views, especially when contrast is used. The base-to-apex tapering pattern, well-illustrated by CMR [25], is also recognizable by echocardiography and helps distinguish true apical hypertrophy from hypertensive remodeling (Figure 10). Among the most promising indices is the indexed apical obliteration ratio, originally defined by Kim et al. [75] in a large echocardiographic cohort. A ratio >0.5 identifies significant apical compartmentalization and correlates with atrial fibrillation, stroke, heart failure and cardiovascular mortality. The absolute length of apical obliteration, with thresholds of >20 mm (high specificity) and >10 mm (overlap with hypertensive cardiopathy) [75], is also measurable with focused apical and short-axis TTE views, particularly when contrast is used (Figure 9). Recent literature has also identified apical papillary muscle displacement as a potential early morpho-functional precursor of ApHCM [76] (Figure 11). Although first described in CMR cohorts, this feature is frequently visible on high-quality TTE and may help identify individuals at risk before the development of overt hypertrophy. Despite the proliferation of apical-centric markers, none of these criteria has yet undergone sufficient large-scale, modality-specific validation, and their transferability to routine TTE varies widely. For this reason, it is essential to organize them within a structured framework that clarifies their level of evidence and practical applicability. Overall, these developments illustrate how CMR-derived geometric and morphologic insights can support a more nuanced and standardized echocardiographic approach to ApHCM. As validation studies expand and multimodality datasets grow, it is likely that selected CMR-derived criteria will progressively inform and refine TTE-based diagnosis, contributing to a more harmonized framework for apical phenotype identification. The 2025 EACVI Clinical Consensus [9] defines the diagnosis of ApHCM not through rigid cut-offs of individual measurements, but rather through a core set of qualitative imaging features such as loss of physiological basal-to-apical tapering, true apical hypertrophy, systolic apical cavity obliteration, mid-ventricular obstruction, apical aneurysm, and typical apical or periapical LGE patterns. Although papillary muscle displacement is not included as a formal diagnostic criterion, the consensus acknowledges apically displaced or morphologically abnormal papillary muscles as a recurrent anatomical feature of the apical phenotype, contributing to its distinctive geometry and potentially influencing distal flow disturbances or mid-cavity obstruction. The consensus does not yet incorporate the newer quantitative apical-centric indices proposed in recent CMR-based studies, such as indexed apical wall thickness, apical angle, and refined apical-to-basal ratios, which remain conceptually aligned with these descriptive features but still require dedicated validation before they can be integrated into formal diagnostic frameworks. Table 2 summarizes the main novel apical-centric diagnostic criteria proposed for ApHCM, including their proposed cut-offs, validation cohorts, strength of evidence, and estimated feasibility in real-world echocardiographic practice.

## 8. What Echocardiography Can Learn from CMRi and Future Perspectives

While CMRi remains the most accurate and comprehensive technique for defining ventricular morphology and tissue characterization, it also serves as the reference standard for research and for the development of new diagnostic criteria. Conversely, TTE represents the first-line imaging tool in clinical practice and therefore must be capable of raising diagnostic suspicion in the majority of cases. The domains in which TTE can derive substantial methodological and conceptual benefit from CMRi can be broadly articulated across different areas. The first domain is operational. The reason CMRi is considered the gold standard for morphological evaluation lies in its intrinsically high signal-to-noise ratio and excellent blood–myocardium contrast, which together provide superior spatial definition. Although such levels of contrast resolution are unlikely to be matched by echocardiography, significant improvement in accuracy can be made by optimizing image acquisition, selecting the best acoustic windows, adjusting parameters to obtain the clearest delineation of the endocardial border, and employing contrast agents to enhance endocardial delineation. The systematic optimization of echocardiographic settings and apical views can therefore minimize foreshortening, improve geometric accuracy, and increase the visibility of apical obliteration or aneurysmal remodeling. The second domain concerns the translation of novel CMR-derived criteria into echocardiographic practice. Even if direct cut-off values identified by magnetic resonance are not yet validated for echocardiography, these criteria can already be applied, at least for a visual/qualitative evaluation to improve morphological interpretation of the apex and guide diagnostic reasoning. Through this conceptual borrowing, echocardiography can progressively align its morphological assessment with that of magnetic resonance, enhancing consistency and diagnostic sensitivity. The third domain is prospective and research-oriented. Future comparative studies between TTE and CMRi may be helpful to evaluate and eventually confirm the concordance and reproducibility of emerging apical-centric indices and to validate their implementation within standardized echocardiographic protocols. A fourth domain lies in the study of intracavitary flow dynamics. The introduction of VFM and BSI now allows TTE to explore intracavitary hemodynamics in ways previously limited to CMRi. These techniques provide quantitative visualization of vortices, flow direction, and energy loss within the ventricular cavity, offering potential functional correlates to the geometric and morphological abnormalities typical of the apical variant. Integration of these approaches may enable the identification of specific flow signatures suggestive of disease even before the development of overt hypertrophy, thereby opening a new frontier in early diagnosis and pathophysiological understanding. The application of CMR-derived apical-centric criteria to TTE must be approached with caution, as none of these parameters have yet been validated through sufficient large-scale, modality-specific studies. Their integration into routine TTE therefore requires a graded and selective interpretation rather than direct adoption. As summarized in Table 2, different CMR-based indices show varying degrees of potential transferability to echocardiography. Some qualitative elements, such as apical obliteration and “spade” appearance, tend to demonstrate a closer alignment between the two modalities and may be more feasible to interpret on TTE, while further validation remains essential, especially regarding quantitative parameters. Other measures, including BSA-indexed apical thickness or the apical angle, appear promising but still require dedicated echocardiographic standardization before they can be reliably incorporated into clinical protocols. Overall, further studies are needed to validate the most promising apical-centric metrics within echocardiography and to assess their reproducibility across different ultrasound platforms and operators. The progressive integration of CMR-informed geometric concepts with echocardiographic strain analysis and emerging flow-based tools (VFM, BSI) may ultimately enhance early detection of apical remodeling and support more refined multimodality risk stratification in ApHCM.

## 9. Clinical Implications

Multimodality imaging in ApHCM has important implications for clinical management, beginning with the diagnostic phase. The combined use of echocardiography and CMRi helps differentiate sarcomeric HCM from infiltrative or metabolic phenocopies. This distinction is clinically relevant, since sarcomeric disease carries different patterns of progression and guides genetic counseling and family screening, while conditions such as amyloidosis or Fabry disease require specific therapies and benefit from early recognition. Imaging also contributes substantially to prognostic assessment. Echocardiography and CMRi together provide the variables needed for the ESC HCM SCD risk calculator, and CMRi refines risk stratification through the quantification of myocardial fibrosis. An LGE burden greater than 15% of LV mass is considered an important risk modifier that may influence ICD indication in selected patients [2,9]. The identification of an apical aneurysm, particularly when associated with extensive LGE, thrombus or significant systolic obliteration, further informs therapeutic decisions and often supports anticoagulation and closer rhythm monitoring [2]. More broadly, multimodality imaging assists in clarifying the presence and significance of ventricular obstruction, guiding individualized medical therapy and, when appropriate, referral to specialized centers. Once the diagnosis of HCM is established, imaging also supports family screening and genetic counseling across all phenotypic variants, enabling early detection and structured follow-up in accordance with current guideline recommendations.

## 10. Conclusions

ApHCM is a distinctive phenotype often underdiagnosed by TTE. CMRi remains the cornerstone of diagnosis, providing superior characterization of morphology, tissue, and perfusion. Beyond its diagnostic role, CMRi serves as a true reference platform from which TTE can learn to provide methodological rigor, quantitative morphology, and tissue-based insight that can be progressively adapted to echocardiographic practice. In fact, CMRi has driven the development of novel diagnostic criteria that hold promise for translation into echocardiographic use. By applying CMRi-derived principles such as apical-centric acquisition, optimized contrast use, and geometric indices, TTE can refine its ability to detect apical hypertrophy and improve diagnostic reproducibility. Although it may never reach the image quality of CMRi, echocardiography can evolve through this process of translational learning, turning research-derived parameters into clinically applicable tools. The integration of novel hemodynamic metrics from 4D-flow CMRi, VFM, and BSI further enhances this cross-modality synergy, consolidating a multimodal and physiologically informed approach to ApHCM where TTE becomes not only complementary to but conceptually enriched by CMRi.

## Figures and Tables

**Table 1:** Comparison between classic HCM and Apical HCM. The apical variant represents a smaller but clinically relevant subset, with distinct epidemiology, imaging challenges, and prognostic factors compared with the classic phenotype. HCM, hypertrophic cardiomyopathy.

Feature	Classic HCM	Apical HCM
**Epidemiology**	Prevalence: 1/500–1/200	1–10% in Europe/North America; up to 25% in Asian cohorts
**Prognosis**	Overall annual mortality ≈ 1%	Overall annual mortality 0.5–3.5%
**Electrocardiographic features**	LV hypertrophy criteria, nonspecific T-wave inversion or repolarization abnormalities	Deep or “giant” negative T waves (10–15 mm)
**Echocardiographic Detection**	Good sensitivity, especially for septal hypertrophy; color-Doppler useful for LVOT obstruction	Often underestimated without contrast; up to 43% of apical aneurysms may be missed by TTE
**Advanced Imaging (CMR** **i** **)**	Confirms diagnosis; differentiates non-sarcomeric causes; quantifies fibrosis (LGE)	Gold standard for apical wall thickness and aneurysm; differentiates non-sarcomeric etiologies; quantifies fibrosis (LGE)

**Table 2:** Overview of the main apical-centric diagnostic criteria proposed for ApHCM, including their proposed cut-offs, validation cohorts, diagnostic accuracy metrics (AUC, sensitivity, specificity, false-positive rate when available), strength of evidence, and estimated feasibility in real-world transthoracic echocardiographic practice. Strength of evidence is a qualitative assessment based on study design, cohort size, degree of external confirmation, and consistency with literature. Feasibility in TTE: “+” = limited or technically demanding; “++” = feasible with careful acquisition; “+++” = readily applicable in routine echocardiographic practice. CMRi, cardiac magnetic resonance imaging; ApHCM, apical hypertrophic cardiomyopathy; AUC, area under the curve; FP, false positive; Sens, sensitivity; TTE, transthoracic echocardiography; HCM, hypertrophic cardiomyopathy; LV, left ventricle.

Criterion	Modality	Cut-Off/Definition	Validation Cohort	Diagnostic Accuracy	Evidence	TTE Feasibility	Reference
**Indexed apical maximum wall thickness**	CMRi	≥5.2–5.6 mm/m^2^	>4000 controls +104 ApHCM pts	AUC 0.94; Sens 99/78%; FP 3%	High	++	Hughes 2024 [25]
**Loss of basal–apical tapering**	CMRi	Apical ≥ basal	22 relative ApHCM vs. controls	Supportive; no AUC	Moderate	+++	Flett 2015 [74]
**Apical–basal thickness ratio**	CMRi	>1 (prev ≥1.3)	Mixed CMR cohorts	Not available	Moderate	+++	Flett 2015 [74]
**Apical angle**	CMRi	≤75–76°	71 pts	AUC 0.77	Low–mod	++	Li 2021 [64]
**Apical thickness progression**	CMRi	Mean ≥7.6 mm; Max ≥9.5 mm	71 pts	AUC 0.87–0.898	Moderate	++	Li 2021 [64]
**Indexed apical obliteration length**	TTE	>0.5	~180–190 ApHCM	Prognostic	Moderate	+++	Kim 2016 [75]
**Absolute apical obliteration**	TTE	>20 mm	~188 ApHCM	Specific	Moderate	+++	Kim 2016 [75]
**Apical papillary displacement**	CMRi	Apical beyond mid-LV	>150 HCM + ctrls	Supportive	Moderate	+	Filomena 2023 [76]
**Apical cavity obliteration severity**	TTE/CMRi	End-systolic closure	Echo/CMR cohorts	Prognostic	Moderate	+++	Hamza 2025 [20]
**Apical aneurysm detection**	CMRi→TTE	Dyskinesis + scar	CMR aneurysm cohorts	Echo misses 43%	High	+	Hamza 2025 [20]

## Data Availability

The examinations from which the images were taken are always available on the portal of the institute to which the corresponding author belongs.

## Abbreviations

The following abbreviations are used in this manuscript:

HCM	hypertrophic cardiomyopathy
LV	left ventricle
ApHCM	apical hypertrophic cardiomyopathy
TTE	transthoracic echocardiography
CMRi	cardiac magnetic resonance imaging
ECG	electrocardiogram
EF	ejection fraction
LVOT	left ventricular outflow tract
MVO	mid-ventricular obstruction
LVOTO	left ventricular outflow tract obstruction
CT	computed tomography
SPECT	single-photon emission computed tomography
PET	positron emission tomography
EACVI	European Association of Cardiovascular Imaging
ASE	American Society of Echocardiography
TEE	transesophageal echocardiography
CW	continuous-wave
GLS	global longitudinal strain
3D	three-dimensional
SSFP	steady-state free-precession
SAM	systolic anterior motion
LGE	late gadolinium enhancement
ECV	extracellular volume
PSIR	phase-sensitive inversion recovery
MBF	myocardial blood flow
MPR	myocardial perfusion reserve
4D-FLOW	four-dimensional flow
VFM	vector flow mapping
BSI	blood speckle
